# Environmental magnetism data of Brantas River bulk surface sediments, Jawa Timur, Indonesia

**DOI:** 10.1016/j.dib.2019.104092

**Published:** 2019-06-03

**Authors:** Mariyanto Mariyanto, Moh Faisal Amir, Widya Utama, Abd Mujahid Hamdan, Satria Bijaksana, Aditya Pratama, Raghel Yunginger, Sudarningsih Sudarningsih

**Affiliations:** a Department of Geophysical Engineering, Faculty of Civil, Environmental and Geo Engineering, Institut Teknologi Sepuluh Nopember, Jl. Raya ITS, Surabaya 60111, Indonesia; b Department of Environmental Engineering, Faculty of Science and Technology, State Islamic University of Ar-Raniry, Banda Aceh 23111, Indonesia; c Faculty of Mining and Petroleum Engineering, Institut Teknologi Bandung, Bandung 40132, Indonesia; d Department of Physics, Faculty of Mathematics and Natural Sciences, Universitas Negeri Gorontalo, Gorontalo 96128, Indonesia; e Department of Physics, Faculty of Mathematics and Natural Sciences, Universitas Lambung Mangkurat, Banjarmasin 70124, Indonesia; f Department of Physics Education, Faculty of Education and Teacher Training, State Islamic University of Ar-Raniry, Banda Aceh 23111, Indonesia

**Keywords:** Environmental magnetism data, Magnetic measurement, Brantas river, Bulk surface sediment, Indonesia

## Abstract

This article presents measurement data using environmental magnetism method on the bulk surface sediments related to the research article entitled “Heavy metal contents and magnetic properties of surface sediments in volcanic and tropical environment from Brantas River, Jawa Timur Province, Indonesia” Mariyanto et al., 2019. Surface sediments were taken from 20 different locations along Brantas River. In the laboratory, a series of magnetic measurements was conducted on sediment samples i.e. magnetic susceptibility, ARM (Anhysteretic Remanent Magnetization) and IRM (Isothermal Remanent Magnetization). These environmental magnetism data were used to characterize bulk surface sediments in the study area.

**Table:** Specifications table

Subject area	Geophysics
More specific subject area	Environmental magnetism
Type of data	Table, graph, figure
How data was acquired	1. Bartington MS2B Susceptibility meter made by Bartington Instrument Ltd., Oxford, UK was used to measure magnetic susceptibility. 2. Molspin AF Demagnetizer made by Molspin Ltd., Newcastle Upon Time, UK was used to apply steady and alternating magnetic field for ARM. 3. An electromagnetic generator was used to apply DC magnetic field for IRM. 4. Minispin Fluxgate Magnetometer made by Molspin Ltd., Newcastle Upon Time, UK was used to measure ARM and IRM intensity.
Data format	Raw
Experimental factors	Surface sediments were sieved (2 mm) then dried at room temperature to produce the bulk samples. All measurements of magnetic susceptibility, ARM and IRM were conducted at room temperature.
Experimental features	Magnetic susceptibility measurement was conducted at dual frequencies (470 Hz and 4700 Hz). ARM measurement was conducted by applying a steady field of 0.05 mT together with an alternating magnetic field of up to 50 mT. IRM measurement was carried out by applying DC magnetic field of up to 1 T.
Data source location	Brantas River, Jawa Timur, Indonesia from Batu city to Mojokerto regency.
Data accessibility	The data are available with this article.
Related research article	M. Mariyanto, M.F. Amir, W. Utama, A.M. Hamdan, S. Bijaksana, A. Pratama, R. Yunginger, S. Sudarningsih, Heavy metal contents and magnetic properties of surface sediments in volcanic and tropical environment from Brantas River, Jawa Timur Province, Indonesia, Sci. Total Environ. 675 (2019) 632–641. https://doi.org/10.1016/j.scitotenv.2019.04.244[1]

**Table:** 

**Value of the data** • Data in this article can be used as a benchmark for the magnetic parameter value of bulk surface sediment from rivers in volcanic and tropical environments and provides information about magnetic characterization. • Data sets can be integrated with other magnetic measurement data such as thermomagnetic and TRM (Thermoremanent Magnetization) for more detailed magnetic characterization. • Data sets can be correlated with chemical content parameters from ICP (Inductively Coupled Plasma) analysis to identify their relationship with rare earth elements.

## 1 Data

In this paper we present detailed data on the environmental magnetism measurement of Brantas River bulk surface sediments [1]. A review has shown recent developments between environmental magnetism with other sciences such as physics, chemistry and biology [2]. Table 1 shows magnetic susceptibility measurement data on Brantas river bulk surface sediment samples. Several other studies have shown that magnetic susceptibility measurement was not only conducted on sediments from rivers [3], [4] but also on sediments from coasts [5], [6] and lakes [7], [8] and other materials such as mineral deposit [9] and guano [10], [11]. Previous studies have shown that frequency-dependent magnetic susceptibility was used to determine superparamagnetic grain content in sediments [12], [13].

**Table 1:** Magnetic susceptibility measurement data of bulk surface sediment samples. χ_lf_ is mass-specific magnetic susceptibility at low frequency, χ_hf_ is mass-specific magnetic susceptibility at high frequency and χ_fd_ is frequency-dependent magnetic susceptibility.

Sample ID	χ_lf_ ( × 10^−8^m^3^kg^−1^)	χ_hf_ ( × 10^−8^m^3^kg^−1^)	χ_fd_ (%)
B1	3163.7	3161.5	0.07
B2	2832.5	2824.3	0.29
B3	4472.8	4344.4	2.87
B4	3164.2	3071.1	2.94
B5	3471.7	3428.3	1.25
B6	3302.8	3213.3	2.71
B7	3782.9	3761.7	0.56
B8	3737.7	3729.2	0.23
B9	4716.3	4667.3	1.04
B10	7231.4	7200.1	0.43
B11	1927.3	1896.9	1.58
B12	1994.1	1975.9	0.91
B13	2385.1	2364.7	0.86
B14	2442.9	2438.2	0.19
B15	3942.3	3925.3	0.43
B16	1753.6	1737.4	0.92
B17	1422.8	1410.2	0.89
B18	1810.4	1806.2	0.23
B19	2059.8	2059.8	0.00
B20	844.0	825.3	2.22
Mean	3022.9	2992.1	1.03
Min	844.0	825.3	0.00
Max	7231.4	7200.1	2.94

**Fig. 1:**
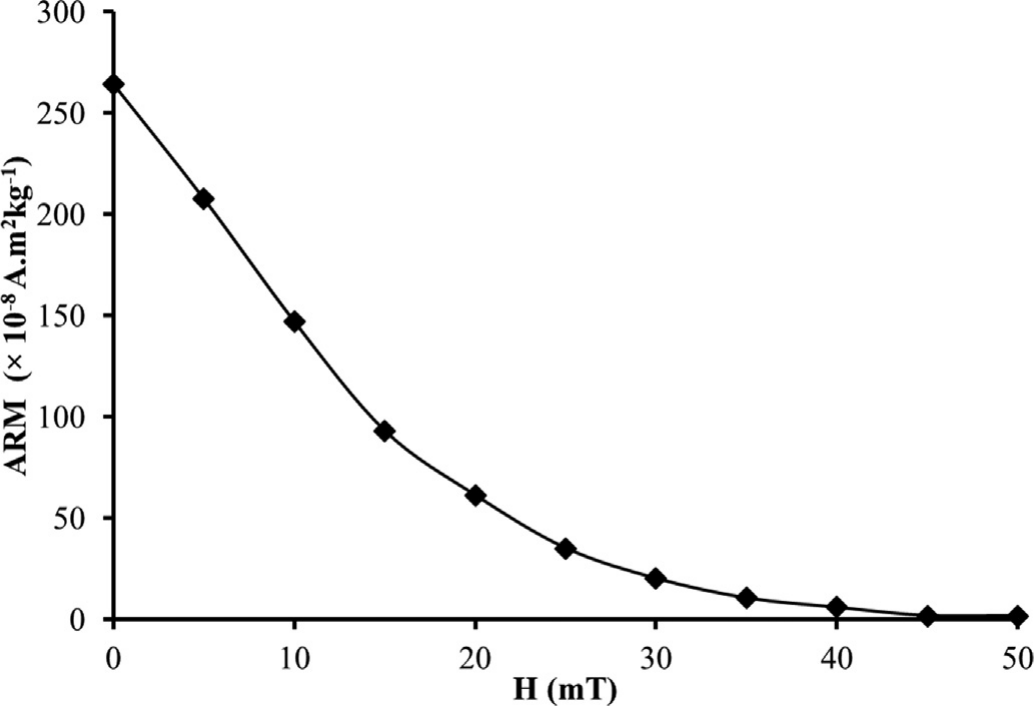
ARM decay curve for typical bulk surface sediment sample (modified after [1]).

The ARM measurement data for representative bulk surface sediment samples are shown in Table 2. Raw data sets for ARM measurements are presented in ".xlsx" format (excel file) in Appendix A. Fig. 1 shows ARM decay curve for typical bulk surface sediment samples. Previous studies showed that ARM measurements were acquired on various samples such as dusts [14], soils [15] and sediments [16], [17] for environmental magnetism studies.

**Table 2:** ARM measurement data of bulk surface sediment sample. N-ARM is Normalized ARM.

H (mT)	ARM Intensity ( × 10^−8^ A.m^2^kg^−1^)	N-ARM
0	264.31	1.00
5	207.73	0.79
10	147.10	0.56
15	93.14	0.35
20	61.33	0.23
25	35.15	0.13
30	20.15	0.08
35	10.51	0.04
40	5.89	0.02
45	1.82	0.01
50	1.81	0.01

**Fig. 2:**
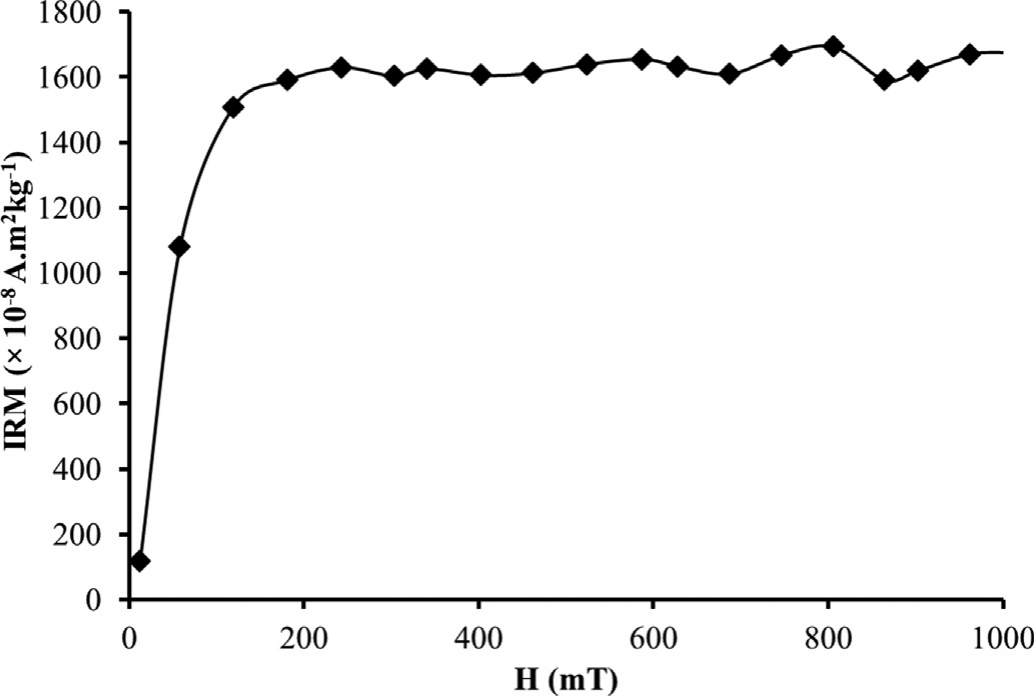
IRM saturation curve for typical bulk surface sediment sample (modified after [1]).

Meanwhile IRM measurement data for representative bulk surface sediment samples are shown in Table 3. The raw data sets for IRM measurements are presented in ".xlsx" format (excel file) in Appendix B. Fig. 2 shows IRM saturation curve for typical bulk surface sediment samples. Previous studies showed that IRM measurements were performed on red clay sediments [18] and loess/paleosol sequence [19], [20] for paleomagnetism studies.

**Table 3:** IRM measurement data of bulk surface sediment sample. N-IRM is Normalized IRM.

H (mT)	IRM Intensity ( × 10^−8^ A.m^2^kg^−1^)	N-IRM
12.02	117.38	0.07
57.77	1080.72	0.64
118.76	1508.53	0.89
181.37	1591.57	0.94
243.17	1630.37	0.96
303.36	1603.46	0.95
341.08	1625.98	0.96
402.88	1606.45	0.95
461.47	1612.76	0.95
524.08	1639.96	0.97
586.68	1655.88	0.98
627.61	1634.08	0.96
687.00	1609.69	0.95
746.40	1667.05	0.98
805.79	1693.62	1.00
864.38	1591.51	0.94
902.10	1619.04	0.96
962.30	1671.35	0.99
1017.67	1675.43	0.99

## 2 Experimental design, materials, and methods

Sampling of surface sediment samples was conducted in 20 different locations along the mainstream of the Brantas river. This river is in Jawa Timur province, Indonesia and passes through several cities or regencies from Batu to Mojokerto. Table 4 shows the locations and coordinates of the sampling sites. Surface sediments were sieved (2 mm) then dried at room temperature to produce the bulk surface sediment samples. They were mashed using a set of mortar and pestle. A cylindrical plastic holder was used to place the samples.

**Table 4:** Detailed locations of the sampling sites along Brantas River.

Sample ID	Geographic Coordinate		Location
	Latitude (S)	Longitude (E)	
B1	7°54′28.627″	112°34′45.423″	Kel. Pendem, Kec. Junrejo, Kota Batu city
B2	7°57′23.127″	112°37′28.957″	Kel. Samaan, Kec. Klojen, Malang city
B3	8°3′37.406″	112°37′52.521″	Ds. Tambaksari, Kec. Tajinan, Malang regency
B4	8°8′24.986″	112°35′10.442″	Ds. Sukorejo, Kec. Gondanglegi, Malang regency
B5	8°8′21.557″	112°27′52.688″	Ds. Sumber Pucung, Kec. Sumber Pucung, Malang regency
B6	8°9′41.870″	112°24′26.225″	Ds. Sukoanyar, Kec. Kesamben, Blitar regency
B7	8°9′55.677″	112°18′28.019″	Ds. Pakel, Kec. Selopuro, Blitar regency
B8	8°9′10.916″	112°13′3.335″	Ds. Satreyan, Kec. Kanigoro, Blitar regency
B9	8°6′57.174″	112°6′11.735″	Ds. Rejotangan, Kec. Rejotangan, Tulugangung regency
B10	8°5′46.375″	112°0′13.735″	Ds. Pulosari, Kec. Ngunut, Tulungagung regency
B11	8°1′6.535″	111°55′32.419″	Ds. Tapan, Kec. Kedungwaru, Tulugangung regency
B12	7°56′13.181″	112°57′22.767″	Ds. Kras, Kec. Kras, Kediri regency
B13	7°51′2.207″	111°59′56.087″	Kel. Manisrenggo, Kec. Kediri, Kediri city
B14	7°44′46.756″	112°1′14.538″	Ds. Gondanglegi, Kec. Prambon, Nganjuk regency
B15	7°40′37.783″	112°4′37.740″	Ds. Papar, Kec. Papar, Kediri regency
B16	7°34′48.551″	112°6′51.674″	Ds. Lestari, Kec. Patianrowo, Nganjuk regency
B17	7°29′30.970″	112°10′3.461″	Ds. Munung, Kec. Jatikalen, Nganjuk regency
B18	7°26′44.020″	112°15′23.150″	Ds. Ngares Kidul, Kec. Gedeg, Mojokerto regency
B19	7°27′23.296″	112°21′22.897″	Ds. Ngares Kidul, Kec. Gedeg, Mojokerto regency
B20	7°26′46.620″	112°27′22.420″	Ds. Mlirip, Kec. Jetis, Mojokerto regency

A series of magnetic measurements i.e. magnetic susceptibility, ARM and IRM was conducted to measure magnetic properties of samples. Measurement of magnetic susceptibility was conducted using Bartington MS2B Susceptibility meter at dual frequencies (470 Hz and 4700 Hz). Measurement of ARM was conducted by applying a steady field of 0.05 mT together with an alternating magnetic field of up to 50 mT using Molspin AF Demagnetizer. Measurement of IRM was carried out by applying DC magnetic field of up to 1 T using an electromagnetic generator. Minispin Fluxgate Magnetometer was used to measure ARM and IRM intensity as the magnetic field changes.
